# Biological Activity Assessment in Mexican Tropical Soils with Different Hydrocarbon Contamination Histories

**DOI:** 10.1007/s11270-015-2621-1

**Published:** 2015-09-29

**Authors:** Jessica Riveroll-Larios, Erika Escalante-Espinosa, Reyna L. Fócil-Monterrubio, Ildefonso J. Díaz-Ramírez

**Affiliations:** Academic Division of Biological Sciences, Academic Group of Evaluation and Environmental Technology, Laboratory of Bioprocess, Juárez Autonomous University of Tabasco, Edif. H. Km 0.5 Carr. Villahermosa - Cárdenas, Villahermosa, Tabasco C.P. 86039 México

**Keywords:** Lipase, Dehydrogenase, Biodegradation, Natural attenuation

## Abstract

The use of soil health indicators linked to microbial activities, such as key enzymes and respirometric profiles, helps assess the natural attenuation potential of soils contaminated with hydrocarbons. In this study, the intrinsic physicochemical characteristics, biological activity and biodegradation potential were recorded for two soils with different contamination histories (>5 years and <1 months). The enzymatic activity (lipase and dehydrogenase) as well as microbiological and mineralisation profiles were measured in contaminated soil samples. Soil suspensions were tested as microbial inocula in biodegradation potential assays using contaminated perlite as an inert support. The basal respiratory rate of the recently contaminated soil was 15–38 mg C-CO_2_ kg^−1^ h^−1^, while the weathered soil presented a greater basal mineralisation capacity of 55–70 mg C-CO_2_ kg^−1^ h^−1^. The basal levels of lipase and dehydrogenase were significantly greater than those recorded in non-contaminated soils (551 ± 21 μg pNP g^−1^). Regarding the biodegradation potential assessment, the lipase (1000–3000 μg pNP g^−1^ of perlite) and dehydrogenase (~3000 μg INF g^−1^ of perlite) activities in the inoculum of the recently contaminated soil were greater than those recorded in the inoculum of the weathered soil. This was correlated with a high mineralisation rate (~30 mg C-CO_2_ kg^−1^ h^−1^) in the recently contaminated soil and a reduction in hydrocarbon concentration (~30 %). The combination of an inert support and enzymatic and respirometric analyses made it possible to detect the different biodegradation capacities of the studied inocula and the natural attenuation potential of a recently contaminated soil at high hydrocarbon concentrations.

## Introduction

Petroleum industry activities such as drilling, transportation and refining in Mexico take place mostly in the southeastern region of the country. Several oil fields are located in the state of Tabasco, where the tropical soils present a variety of edaphic and ecological characteristics (Adams et al. 2009; Díaz-Ramírez et al. 2013). Documented oil spills in this region have affected large areas of soil, rivers, streams and lagoons. Some of these spills occurred more than 20 years ago, but others are much more recent (Adams et al. 2012; Cram et al. 2004; Gallegos Martínez et al. 2000). Recently, contaminated sites are reclaimed by applying active bioremediation strategies, such as land farming, biostimulation-bioaugmentation, biopiles and soil washing with biosurfactants (Medina-Moreno et al. 2011; Vyas and Dave 2011), as an immediate response to the environmental emergencies caused by oil spills. In contrast, if the presence of hydrocarbons and the environmental conditions (flood-drought cycles, extreme temperatures, leaching and increased solar radiation) result in a weathered contaminated soil, in which the residual hydrocarbons are more complex, recalcitrant and less bioavailable to the native microbial populations (Maletić et al. 2011). In these cases, passive methods (natural attenuation, phytoremediation, biostabilisation) are viable alternatives for the self-recovery of the sites, especially those with a high ecological and agricultural value (Adams et al. 2011). Usually, physicochemical parameters are used (organic matter, nutrient balance, water retention capacity, repellency, etc.) to asses soil quality and establish a remediation end point (Adams et al. 2008); however, as they change slowly, long periods are required to identify significant changes. Other important criteria when selecting remediation technologies and carrying out monitoring in the field, due to their quick response, are the microbial activity levels in response to the presence of hydrocarbons and the weather conditions that prevail at a site (Margesin et al. 2007; Riffaldi et al. 2006). Biological indicators such as the type and number of predominant microorganisms, enzymatic activities (dehydrogenases, lipases, oxygenases) and degradation kinetics (measured in the field or the laboratory), as well as the half-life of the contaminants, have been analysed successfully in order to determine the level of microbial activity in contaminated soils, to evaluate the biodegradation of the contaminants by the microbial community and to determine the resulting benefits after treating the soil (Liu et al. 2011; Chang et al. 2010; Bending et al. 2004). Data from microbial activities and biodegrading potential, related to contamination soil histories, are relevant because they are useful to diagnose bioremediation feasibility and to determine end points after remediation strategies. Our aim was to evaluate biological indicator levels and the biodegradation potential of native microbial inocula in tropical soils with different hydrocarbon contamination histories. The analysis was based on the enzymatic activity (lipase and dehydrogenase) and respirometric profiles (mineralisation) recorded throughout biodegradation assays carried out under non-limiting hydrocarbon availability conditions.

## Materials and Methods

### Study Area

The study area is located in the state of Tabasco, southeastern Mexico. The contaminated sites are adjacent to facilities with oil activities. The first site is located in Villa La Venta, near a petroleum refining facility, with clay soil and mangrove vegetation, where hydrocarbon spills have historically occurred (Pérez-Cadena 2010). The second, a recently contaminated site (<1 month), is located in the Samaria-Luna oil extraction field, in the Cunduacán municipality. The sites were selected based on a comparative analysis of the type and concentration of contaminants, time of contamination, actual state of the contaminated sites, access availability to the sites, vegetation and the presence of water bodies.

### Soil Sampling

Soil samples were collected with a hand auger and spatula, following the Mexican regulations as technical guideline (NOM-138-SEMARNAT/SS-2003). The sampling points were selected considering the level of contamination and the characteristics of the soil after a visual inspection. Four samples were collected at the weathered site and three samples at the recently contaminated site. Three types of samples were collected at both sites: (a) for a physicochemical analysis (2.5 kg soil) in black plastic bags (hydrocarbon content), (b) for microbiological and enzymatic analyses (200 g soil) in glass bottles with a Teflon septa, and (c) for biodegradation assays (2 kg soil) in black plastic bags. Samples were collected from two strata: 0–10 cm and 10–45 cm. Once labelled and registered, the samples for the microbiological analyses and the enzymatic activity quantification were transported (at room temperature) to the Bioprocess Laboratory for processing. The samples for the enzyme determination were kept at room temperature in order to minimise disruption of the natural activity of the native microorganisms. Before carrying out the corresponding analyses, the moist soils were sieved (4.7 mm) to remove stones and plant debris (roots), and to homogenise the particle size.

### Media Culture and Support (Perlite)

Biodegradation assays and microbial recording were carried out in a mineral base medium (MBM1) with the following composition (g l^−1^): 3.0 NaNO_3_, 0.30 MgSO_4_·7H_2_O, 0.50 KCl, 13.0 KH_2_PO_4_ and 1.0 K_2_HPO_4_. To this was added Na_4_P_2_O_7_·10H_2_O (1.80 g) to prepare the soil suspension (microbial inocula, MBM2). Both media were adjusted to pH 6 with 2 N NaOH (Díaz-Ramírez et al. 2008).

The biodegradation assays were carried out using perlite as a support and light Olmeca Crude Oil as a carbon source. The perlite was sieved (2.8 mm), washed thrice with hot distilled water (60 °C) and dried in an oven at 105 °C for 48 h. The Olmeca Crude Oil was heated to eliminate volatile compounds (5 days at 70 °C). Oil-spiking was later carried out under manual mixing, using *n*-hexane as a carrier. The resulting support was allowed to evaporate under a fume hood for 48 h.

### Basal Activity in the Soil

The soil samples were manually mixed and stored to be analysed 48 h after sampling. The following physicochemical parameters were recorded: (a) pH, (b) conductivity (dS m^−1^), (c) humidity (%) and (d) total hydrocarbon content (mg TPH kg^−1^). All samples used for the analyses were previously dried. At the same time, the following biological and microbiological indicators were analysed: (a) basal respiration (mg C-CO_2_ kg 24 h^−1^), (b) lipase enzymatic activity (μg pNP kg^−1^ 10 min^−1^), (c) dehydrogenase enzymatic activity (μg INF kg^−1^ day^−1^), (d) microbial total count (CFU g^−1^) and (e) hydrocarbon-degrading microorganisms according to the MPN method (cell number g^−1^).

### Biodegradation Potential Assays with Differently Contaminated Soils

#### Soil Suspension Preparation

Recently contaminated soil (RCSS) and weathered soil (WSS) suspensions were prepared by stirring (4 h) contaminated soil samples (100 g dry weight) in flasks containing 750 ml of MBM2 at 30 °C. Volumes of 15 ml of the resulting soil suspensions were transferred to the experimental units containing perlite for the biodegradation assays.

#### Biodegradation Assays

Biodegradation kinetic assays were carried out following a completely randomised experimental design with the factors: (a) inoculum source (RCSS and WSS), (b) hydrocarbon concentration (10,000 and 20,000 mg kg^−1^, and a non-contaminated control) and (c) incubation time. Each experimental unit (500 ml glass bottles) contained perlite (10.2 g), MBM1 (10 ml), sterile distilled water (5 ml) and SS as the inoculum (15 ml). The experiment setting comprised a group of 15 bottles, separated into five bottles per treatment. Nine bottles were connected to a SS4 respirometry system to continuously analyse the produced CO_2_. The remaining six bottles were incubated under similar conditions (water bath at 35 °C) for 30 days. In order to maintain the field capacity of the perlite around 60 %, the water lost by natural evaporation was replaced every week.

One bottle per treatment was selected for sampling every 7 days during the incubation period to determine the lipase and dehydrogenase enzymatic activities. Samples were subdivided to obtain nine sub-samples per treatment (0.5 g). The variables evaluated throughout the experiment were (a) lipase and dehydrogenase enzymatic activity, (b) biodegradation, (c) produced CO_2_ and (d) initial and final total microbial count (CFU g^−1^ of perlite).

### Hydrocarbon Analyses

The residual hydrocarbon content was gravimetrically measured after its extraction from the solid samples following the Soxhlet method (EPA 3540 method; NOM-138-SEMARNAT/SS-2003) and using a mixture of acetone/hexane (1:1) as solvent. Oil concentrates were recovered after solvent separation using a rotary evaporator (Büchi model R-210).

### Respirometric Analyses

A respirometric system was used (Sable Systems SS4®) in order to record the production of CO_2_ throughout the biodegradation assays. The system was designed as a pushed open system with a mass flow control valve and automated sampling. The system makes it possible to record CO_2_ at different recording cycles according to the culture stage (4, 6, 8, 12 and 24 h). The sensor base line was adjusted to zero with a CO_2_-free air inlet after every recording cycle. The raw data of the produced CO_2_ of all the samples were transformed to obtain volumetric data (ml min^−1^) and, later, mass units (mg C-CO_2_ kg^−1^ perlite) using the ExpeData software (Sable Intl. Co.). Finally, the accumulated produced CO_2_ and the production rates (mg C-CO_2_ kg^−1^ perlite h^−1^) were calculated from the raw data.

### Enzymatic Analyses

#### Dehydrogenase

The method for measuring DHS activity was adapted from those proposed by Von Mersi and Schinner (1991) and Neto et al. (2007). Iodo-nitro-tetrazolium (5 mM INT) was used as electron acceptor in the presence of Tris buffer (1 M, pH 7) in each soil or perlite sample. The produced red formazan was extracted with acetone (Baker) from the samples after being incubated for 24 h at 30 °C. It was later quantified spectrophotometrically (*λ* = 493 nm) using a standard curve at different concentrations (5, 10, 15, 20, 25, 30 and 35 μg INF ml^−1^ acetone). When necessary, samples were diluted with acetone.

#### Lipase

Lipase activity was recorded following the method proposed by Margesin (2005). Soil samples were incubated in the presence of *p*-nitrophenyl-butyrate (pNPB) as substrate and a phosphate buffer solution (pH 7). The produced *p*-nitro-phenol was spectrophotometrically quantified (*λ* = 400 nm) using a standard curve at different concentrations (0, 25, 50, 75, 100 and 125 μg pNP 5 ml^−1^ buffer solution). When necessary, samples were diluted with the same buffer.

### Microbial Analyses

Total counts of the bacterial and fungal populations were recorded following Lorch et al. (1995) on trypticasein soy agar (TSA; Bioxon, Mexico) and potato dextrose agar (PDA; Bioxon, Mexico), respectively. Cultures were incubated at 30 °C and counts were carried out after incubation periods of 2 days (bacterial) and 3 to 5 days (fungal). The number of microorganisms able to grow in TPH (500 mg l^−1^) as the sole carbon source was assessed by the most probable number (MPN) method, based on the procedure reported by Wrenn and Venosa (1996). The samples were incubated for 2 weeks at 30 °C. Later, iodo-nitro-tetrazolium (Sigma Chemical Co., St. Louis, Mo.) at 535 μg ml^−1^ was used as an indicator. The number of hydrocarbon degrading microorganisms was calculated using the MPN-calculator (EPA V 4.04).

### Statistical Analyses

The recorded variables were analysed statistically with the Stat Graphics 5.1 software package. An analysis of variance (ANOVA) was carried out for the basal assays. A multivariate ANOVA was carried out for both the enzymatic and respirometric activities to determine differences between treatments (hydrocarbon concentration) and inocula (different contamination history).

## Results and Discussion

### Hydrocarbon Contaminated Soil Characterisation (Physicochemical and Biological Soil Characterisations)

#### Weathered Soil

Three sampling areas were selected near the refining facilities in La Venta: (a) a vegetated flooded area, (b) the wall of a drain facing a mangrove forest and (c) a highly contaminated area adjacent to the drain. Three soil samples were collected and physicochemically characterised (Table 1). The soils at these sites presented a visual and textural aspect typical of weathered hydrocarbon contaminated soils (dark colour, intense oil smell, sticky consistency). They were classified as strongly acidic (pH 3.9–5.2) and slightly saline (0.92 to 1.56 dS m^−1^). The organic matter in the soils was three times greater than the recently contaminated soil, possibly resulting from the abundant vegetation and the frequent crude oil inputs. However, other parameters associated with soil fertility, including cationic exchange capacity (Table 1) and salinity, indicated the adverse effects of the complex and high molecular weight hydrocarbons. Previous studies on this type of soil have reported a high repellency and a loss of water retention capacity (Adams et al. 2008). Most of the weathered soils presented a texture corresponding to sandy-loam (81–85 % sand content), except for sample SLV-P9M9 which was clay-loam and was collected at a contaminated point adjacent to the mangrove forest near the drain. The TPH content (~77,000 mg kg^−1^) recorded in the weathered soil samples was greater than the concentration (~20,000 mg kg^−1^) registered in the recently contaminated soil samples. Soil samples collected from areas with similar environmental and geomorphological conditions (high TPH levels, flooded clay soils) have been studied previously (Adams et al. 2009; Cram et al. 2004; Gallegos Martínez et al. 2000; Zavala et al. 2005).

**Table 1:** Recently contaminated soil and weathered soil characterisation

Soil ID	SOM^a^	N_t_	P Olsen	K	Ca	Mg	Na	CEC^b^	TPH^c^	Clay	Silt	Sand	Textural class
	%	mg kg^−1^				cmol kg^−1^			mg kg^−1^		%		
Recently contaminated soil													
SSL-C1	8.67	0.17	1.59	0.02	ND	1.28	1.33	29.74	17,207 ± 204	72	13	15	Clayed
SSL-C2	4.67	0.12	0.87	0.02	ND	5.44	6.98	27.06	20,395 ± 315	63	10	27	Clayed
SSL-C3	5.5	0.26	12.4	0.08	1.45	6.17	0.53	28.28	12,211 ± 192	62	31	7	Clayed
Weathered soil													
SLV-P1M1	27.67	0.29	32.8	0.02	ND	2.40	9.29	8.29	77,098 ± 638	10	9	81	Sandy-loam
SLV-P1M3	23.67	0.20	41.4	0.02	ND	2.34	8.95	7.31	64,548 ± 523	10	7	83	Sandy-loam
SLV-P7M7	17.17	0.20	19	0.01	ND	1.87	8.81	4.88	37,735 ± 702	8	7	85	Sandy-loam
SLV-P9M9	14.34	0.34	6.67	0.06	2.62	10.02	21.83	22.43	55,670 ± 603	34	33	33	Clay-loam

The TPH content and the physicochemical characteristics were recorded following Mexican regulations as guidelines (NOM-138-SEMARNAT/SS-2003; NOM-021-RECNAT-2000)

*ND* not detected (below the detection limit)

^a^Soil organic matter recorded following the Walkley and Black method (NOM-021-RECNAT-2000)

^b^Cation exchange capacity

^c^Total petroleum hydrocarbon

#### Recently Contaminated Soil

Recently contaminated soil was collected 1 month after a major oil spill (Olmeca crude oil). Four sampling areas were selected: (a) an oil-impregnated pasture where spilled oil accumulated, (b) the containment wall near a non-contaminated area, (c) a point near the access to the site where oil accumulated and (d) a point adjacent to the pipeline (drain) on a sandy shore. Nine soil samples were collected and characterised. All samples had a neutral pH (~7.04–7.92) and an average conductivity characteristic of non-saline soils (~0.65 dS m^−1^). The resulting SOM (soil organic matter) and CEC (cation exchange capacity) corresponded to soils used for cattle grazing and grassland soil health. The soil texture analysis classified it as sandy-loam. Considering the short time between the spill and the sampling, it is likely that these soil properties, at the time, still remain without major alterations.

### Basal Biological Activity

The effect of the hydrocarbon contamination history on the microbial activity in the soil was evaluated. The analyses were based on the microbial composition and the quantification of the enzymatic (lipase and dehydrogenase) and respirometric (background) activities in recently contaminated and weathered soil samples. The resulting profiles were used to select the soil inocula used for assaying the biodegradation potential of these soils under non-bioavailability limitations.

#### Heterotrophic and Hydrocarbon-Degrading Microorganisms

Soil samples from the two contaminated sites were microbiologically characterised following conventional methods. The results are presented in Table 2. The bacterial counts in the weathered soil samples growing on TSA were greater (1.2–23 × 10^7^ CFU g^−1^) than those recorded in the recently contaminated soil samples (~1.2 × 10^7^ UFC g^−1^). The fungal counts growing on PDA were twice smaller than the bacterial numbers and were similar in both types of soil (~0.21–4.8 × 10^5^ CFU g^−1^). The hydrocarbon-degrading microbial counts were greater in the weathered soil (5 × 10^7^–4.4 × 10^9^ cell g^−1^ of soil) than in the recently contaminated soil (0.2–9.4 × 10^8^ cell g^−1^ soil) (Table 2).

**Table 2:** Total counts of bacterial and fungal populations and TPH degrading microorganisms count for the recently contaminated soil and weathered soil samples

Soil sample	Total bacterial count^a^		Degrading-hydrocarbons microorganism count^b^
	Bacteria	Fungi	Cell number × 10^6^ g^−1^
	CFU × 10^7^ g^−1^	CFU × 10^5^ g^−1^	
Recently contaminated soil			
SSL-1	>10.00	>10.00	24.20
SSL-2	1.16 ± 0.01	4.83. ± 0.25	946.72
SSL-3	1.20 ± 0.01	0.88 ± 0.01	123.92
Weathered soil			
SLV1	0.10 ± 0.01	0.78 ± 0.01	1.10
SLV2	0.13 ± 0.01	0.26 ± 0.00^c^	0.72
SLV3	1.22 ± 0.38	0.21 ± 0.00^c^	49.62
SLV4	13.30 ± 2.50	2.00 ± 0.02	10.32
SLV5	23.00 ± 0.25	3.83 ± 0.00^c^	4,409.96
SLV6	0.46 ± 0.00^c^	0.45 ± 0.00^c^	ND^d^

^a^Values obtained for three replicates per dilution. Counts greater than 10 × 10^7^ are indicated as >10.0

^b^Values were calculated using the MPN Calculator software, version 4.04 (Klee 1996. EPA)

^c^Standard deviation <0.003

^d^Non-determined soil sample

#### Soil Respirometry

**Fig. 1:**
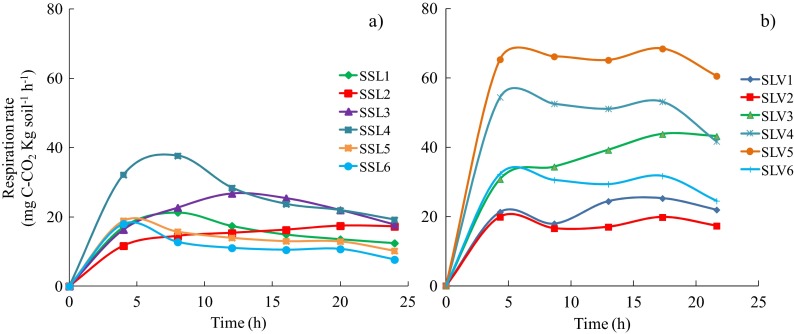
CO_2_ production rate (mg C-CO_2_ kg^−1^ h^−1^) in samples of **a** recently contaminated soils and **b** weathered soils. The values were obtained during a 24-h kinetic cycle

In order to assess the intrinsic respiratory activity in several soil samples, the production of CO_2_ under controlled conditions was recorded during a 24-h kinetic cycle. The resulting CO_2_ production rate profiles are presented in Fig. 1a and b. Samples from the recently contaminated soil presented similar profiles with an average of 20 mg C-CO_2_ kg^−1^ h^−1^ (Fig. 1a). The controlled soil respiration assay indicated that the maximum production rates for most of the samples were registered after 8 h and remained throughout the time window analysis.

The maximum CO_2_ production rate of 38 mg C-CO_2_ kg^−1^ h^−1^ was recorded for sample SSL4 (Fig. 1a). For the weathered soil, the CO_2_ production rates were markedly different among the samples (Fig. 1b). The greatest average CO_2_ production rates were recorded for samples SLV4 and SLV5, ranging from 55 to 70 mg C-CO_2_ kg^−1^ h^−1^. In contrast, the basal soil respiration in a non-contaminated soil collected near the contaminated site varied from 1.02 to 1.08 mg C-CO_2_ kg^−1^ h^−1^. Guzmán-Osorio (2011) reported a basal soil respiration of 1.4 mg C-CO_2_ kg^−1^ h^−1^ for soil in a flooded pasture in the southeastern region of the state of Veracruz (near the contaminated site in Tabasco). Similar results were obtained by Sánchez et al. (2005) of around 1.9 mg C-CO_2_ kg^−1^ h^−1^ for soil in a tropical savannah.

For sample SLV5, the level of CO_2_ production (68.38 mg C-CO_2_ kg^−1^ h^−1^) was positively correlated with the high hydrocarbon-degrading number (Table 2), reaching a maximum activity 21 h after the assay started. Soil respiration and nutrient and microorganism content have been used to estimate biological activity in the presence of organic pollutants, such as petroleum hydrocarbons (Supaphol et al. 2005). The respirometric results were used as background mineralisation (basal respiration) to select the soil samples most suitable for the biodegradation potential assessment.

#### Lipase and Dehydrogenase Enzymatic Activity

##### *Lipase*

**Fig. 2:**
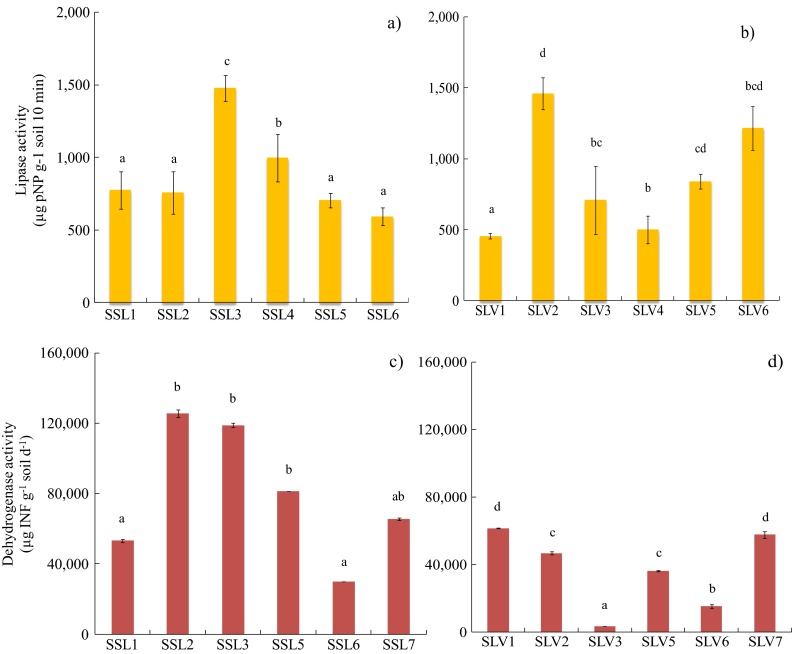
Lipase (μg pNP g^−1^ soil 10 min^−1^) and dehydrogenase (μg INF g^−1^ soil 24 h^−1^) activities recorded in **a** lipase in recently contaminated soil, **b** lipase in weathered soil, **c** dehydrogenase in recently contaminated soil and **d** dehydrogenase in weathered soil. The *error bars* represent the standard deviation of three replicates. The letters *a*, *b*, *c* and *d* indicate the statistical difference among samples

Lipase activity in the soil was recorded in all the samples collected from the different contaminated sites. The results may be seen in Fig. 2a and b. The greatest lipase activity was recorded in sample SSL3 (1477.5 mg pNP g^−1^ 10 min^−1^) (Fig. 2a), followed by sample SSL4 (996.0 mg pNP g^−1^ 10 min^−1^). Statistical analyses showed significant differences (*p* < 0.05) between the most active samples and the other recently contaminated soil samples (~600 mg pNP g^−1^ 10 min^−1^). Samples from the non-contaminated soil adjacent to the contaminated site presented a lipase activity of 531.2 ± 37.4 mg pNP g^−1^ 10 min^−1^. These results reflect the induction of the lipases due to the presence of the TPH mixture, composed mostly of *n*-alkanes of medium carbon chain, which are easily assimilated by soil microorganisms that produce metabolites that provide a substrate for microbial hydrolases. Additionally, the high levels of lipase activity in the recently contaminated soils agree with the respiration rates (Fig. 1a) measured in the short test for the corresponding samples (i.e. SSL3 and SSL4).

Regarding the weathered soil samples (Fig. 2b), two samples (SLV2 and SLV6) presented the greatest lipase activity with values of 1200 to 1461.02 mg pNP g^−1^ 10 min^−1^. Significantly lower lipase activity values (500 to 700 mg pNP g^−1^ 10 min^−1^) were recorded in most of the weathered soil samples (Fig. 2b).

Lipase activity in most of the samples from the two sites was above 500 μg pNP g^−1^ 10 min^−1^, which is considerably greater than the values reported for most contaminated soils. Díaz-Ramírez et al. (2010) studied the effect of hydrocarbon concentration on microbial activity through the application of different bioremediation treatments, and recorded average lipase values below 400 μg pNP g^−1^ 10 min^−1^ for biostimulated soils (10,000 mg kg^−1^) and around 240 μg pNP g^−1^ 10 min^−1^ for non-contaminated clay-loam soil, after 30 days of treatment. In general, the lipase enzyme has been used as a biological indicator for hydrocarbon biodegradation as it initiates lipid decomposition (glycerol ester hydrolases), which acts on the carboxyl ester bondings in acylglycerol to produce a release of fatty acids and glycerol (Margesin et al. 2007; Riffaldi et al. 2006; Sirisha et al. 2010). Lipases have broad substrate specificity and are active throughout a wide range of temperatures. They are involved in diverse biotechnological applications because of their biocatalytic versatility (Hasan et al. 2009; Joseph et al. 2008).

##### *Dehydrogenase*

Dehydrogenase activity was recorded in the same soil samples collected at the recently contaminated site and the weathered site (results in Fig. 2c and d). For the recently contaminated soils, the greatest dehydrogenase activity was recorded in sample SSL2 (~125,560.7 μg INF g^−1^ soil 24 h^−1^) (Fig. 2c), followed by samples SSL3 and SSL5 (80,000 to 100,000 μg INF g^−1^ soil 24 h^−1^). These dehydrogenase levels were significantly greater than those recorded in samples SSL1, SSL6 and SSL7. In contrast, the weathered soils presented a maximum dehydrogenase activity of 46,800 to 61,450 μg of INF g^−1^ of soil after 24 h of incubation (SLV1, SLV2, SLV5 and SLV7). However, the dehydrogenase activity values of both soils indicate a high oxidative microbial metabolism. The higher DHS values observed in the recently contaminated soil (Fig. 2) may be attributed to the presence of easily biodegradable compounds like *n*-alkanes (C10-C21) and low molecular weight aromatic compounds which are the main component of the light crude oil spilled at the site. In contrast, in the weathered soil the residual hydrocarbon is composed mostly of more recalcitrant fractions (Acosta and Paolini 2005; Gallegos Martínez et al. 2000). Mirás Avalos et al. (2007) found a strong positive correlation between dehydrogenase (DHS) and respiratory activity (CO_2_ production), used as microbial activity indicators in eroded soils. These results are similar to those obtained for the hydrocarbon-contaminated soils collected in the present study at the highly contaminated site at La Venta. The soil respiration recorded in sample SLV6 was 68.38 mg C-CO_2_ kg^−1^ h^−1^, which corresponds to one of the higher DHS activities and could be associated with a high degrading-microorganism number in this sample (Table 2).

### Biodegradation Potential Assays

A biodegradation assay was designed to determine the biodegradation efficiency of the microbial community composing the soil inocula obtained from the two sites with different contamination histories. The assay was carried out using perlite as an artificially contaminated support (~10,000 and 20,000 mg kg^−1^) to simulate non-bioavailability hydrocarbon limiting conditions. The recorded biological indicators were the lipase and dehydrogenase activities, along with respirometric profiles (evolved CO_2_) in response to high hydrocarbon concentrations.

#### Lipase Activity

**Fig. 3:**
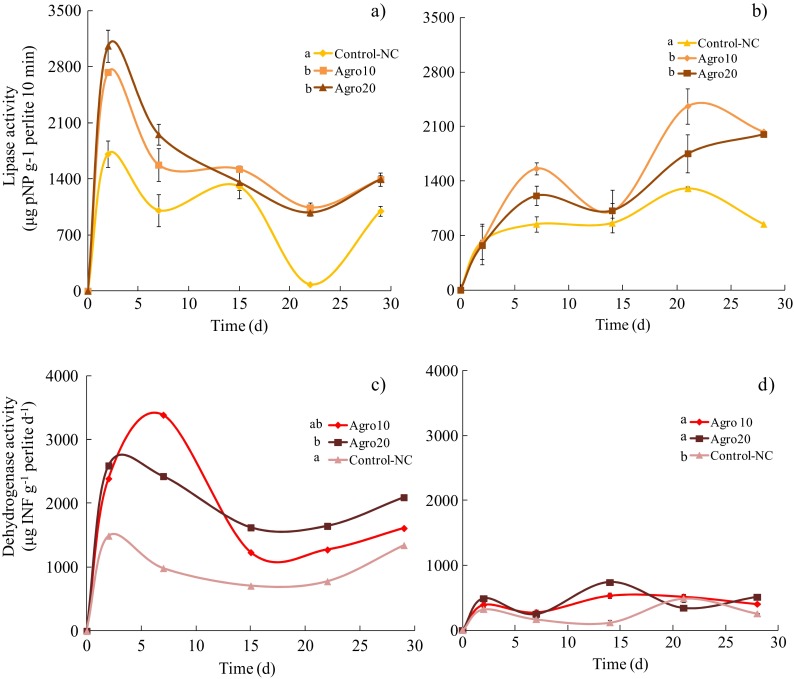
Lipase and dehydrogenase kinetics recorded in contaminated perlite (0, 10,000, 20,000 mg kg^−1^). Lipase activity in the inocula of the recently contaminated soil suspension and the weathered soil suspension is presented in panels (**a**) and (**b**). Dehydrogenase activity in the two inocula is presented in panels (**c**) and (**d**). The *error bars* represent the standard deviation of nine sub-samples for each replicate. The letters *a*, *ab* and *b* indicate the statistical difference between the two inocula (*p* < 0.05)

The lipase activity recorded in the recently contaminated soil (RCSS) and the weathered soil (WSS) suspensions is shown in Fig. 3a and b. The lipase activity in the RCSS increased with respect to the hydrocarbon concentration, reaching a maximum of 3000 μg pNP g^−1^ perlite in 20,000 mg kg^−1^ of hydrocarbons, and 2700 μg pNP g^−1^ perlite in 10,000 mg kg^−1^ of hydrocarbons (Fig. 3a). The greatest values were recorded during the first 7 days of the culture in all the treatments. After this period, the lipase activity decreased to around 1400 μg pNP g^−1^ perlite for both hydrocarbon concentrations. The lipase activity in the WSS increased gradually up to a maximum at day 7 (Fig. 3b), with slightly greater values in the lower hydrocarbon concentration (~1400 μg pNP g^−1^ perlite) than those recorded in the higher concentration. After this period, the lipase activity increased throughout the assay to reach another maximum at day 21 of incubation (Fig. 3b), with values of 2362.42 ± 277 and 1753.01 ± 96 μg pNP g^−1^ perlite in 10,000 and 20,000 mg kg^−1^ of hydrocarbons, respectively. The lipase activity was significantly greater when the RCSS was used as the inoculum than when the WSS was used. The lipase activity in the control bottles (non-contaminated perlite) was lower than that recorded in the treatments with hydrocarbons and in those inoculated with RCSS and WSS (Fig. 3a, b). Statistical analyses of the enzyme activity values, using a Kruskal-Wallis test, revealed significant differences (*p* < 0.05) between the inocula (RCSS and WSS) and between the contaminated and non-contaminated perlite in each assay.

Lipase activity that responds to the presence of hydrocarbons during different bioremediation treatments has been studied (Maila and Cloete 2005; Margesin and Schinner 2001; Margesin et al. 2007), and stable values have been recorded even with low rates of hydrocarbon biodegradation. Margesin et al. (2000) reported low levels and a fast decrease in lipase activity throughout the monitoring time in a field study of aged biodiesel contaminated soils. The composition and recalcitrance of the residual compounds were identified as factors that may influence lipase activity in aged contaminated soils. Nonetheless, the monitoring of lipase activity has been successfully used as a biological indicator. In a study of soils with different TPH concentrations and input of nutrients, Margesin et al. (2007) reported a parallel behaviour between TPH removal and lipase activity, indicating an increase in the enzyme together with an increase in hydrocarbon loading rates. Lipase activity behaves similarly under non-limiting bioavailability conditions, such as those established in the present study, with high levels in contaminated perlite and in the inocula obtained from both the recently contaminated soil and the weathered soil. A negative correlation between enzyme activity and TPH content was recorded in all the treatments, indicating that the greatest lipase activity coincided with the lowest hydrocarbon content. The induction of the enzyme has been proposed (Margesin 2005) and may respond to the presence of hydrocarbon biodegradation sub-products, which are substrates for lipases. This enzymatic response makes it possible to detect the high hydrocarbon-biodegrading capacity of the microbial community present in the recently contaminated soil.

#### Dehydrogenase Activity

The dehydrogenase activity recorded throughout the biodegradation potential assays for the RCSS and WSS suspensions is presented in Fig. 3c and d. Dehydrogenase levels in the presence of the RCSS inoculum increased during the first 7 days of the culture, reaching a maximum of 3390 μg INF g^−1^ perlite 24 h^−1^ in 10,000 mg kg^−1^ of perlite (Fig. 3c) and 2560 μg INF g^−1^ perlite 24 h^−1^ in 20,000 mg kg^−1^ of perlite in the experimental bottles. Background levels of the enzyme (non-contaminated control) were significantly lower (708–1500 μg INF g^−1^ perlite 24 h^−1^) than those recorded in the contaminated perlite (Fig. 3c), probably due to the presence of residual organic matter and hydrocarbons in the soil suspensions used as inocula.

The dehydrogenase activity in the perlite inoculated with the WSS inoculum is presented in Fig. 3d. The enzymatic activity remained below 1000 μg INF g^−1^ perlite 24 h^−1^ in all the treatments. The maximum activity (~800 μg INF g^−1^ perlite 24 h^−1^) was recorded in the contaminated perlite with 20,000 mg kg^−1^ after 15 days (Fig. 3d). In general, the dehydrogenase activity was significantly greater (three times) in the perlite inoculated with the recently contaminated soil suspension than with the weathered soil suspension (Fig. 3c, d).

Furthermore, the Kruskal-Wallis statistical analyses indicated significant differences (*p* < 0.05) in the enzymatic activity among the inocula, with greater values in the RCSS (3388.37 μg INF g^−1^). With respect to hydrocarbon concentration, the RCSS presented significant differences (*p* < 0.05) among all the treatments (10,000 and 20,000 mg kg^−1^, and the control), indicating that the initial concentrations produce an increase in enzymatic activity at a greater concentration. Nonetheless, the WSS presented differences in enzymatic activity between the contaminated perlite (10,000 and 20,000 mg kg^−1^) and the control.

Dehydrogenase activity has been linked to the first stages of the aerobic metabolic activity during the biodegradation of different organic compounds, such as aliphatic and aromatic hydrocarbons. This could explain the low activity of this enzyme in the weathered soil, where residual compounds are mostly constituted by heavy fractions like resins and asphaltenes (Díaz-Ramírez et al. 2003; Escalante-Espinosa et al. 2005), instead of medium carbon chain compounds (C10–C18) which typically compose the TPH (light and medium fractions), like those used in the perlite assays. The importance of monitoring and understanding the activity of the oxidoreductases in the soil lies in the fact that processes like mineralisation and organic matter humification are led mainly by oxidation, reduction and hydrolysis reactions (Pascual et al. 2000; Van Hamme et al. 2003), processes that are important in the biodegradation of organic compounds, including complex mixtures (TPH).

## CO_2_ Production and Hydrocarbon Biodegradation

Microbial respiratory activity is a good indicator of the biodegradation of organic compounds and substrate mineralisation, as it is linked to microbial growth. A respirometric assay was thus designed to determine the CO_2_ production rate and the cumulative mineralisation through automated measurements, in closed bottles, with contaminated perlite and RCSS and WSS as inocula.

### Recently Contaminated Soil Inoculum

**Fig. 4:**
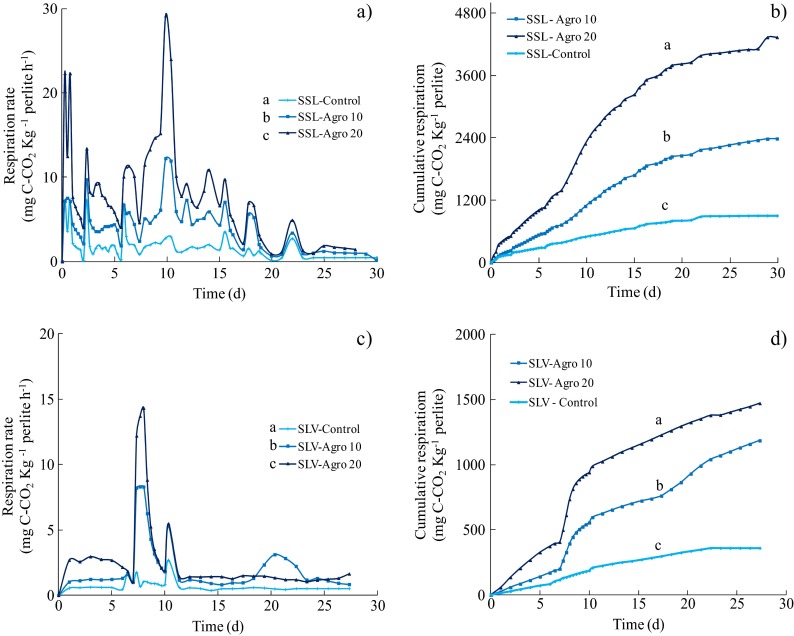
CO_2_ production rates (mg C-CO_2_ kg^−1^ perlite h^−1^) and cumulative CO_2_ production profiles recorded in contaminated perlite (0, 10,000, 20,000 mg kg^−1^) inoculated with the recently contaminated soil suspension (**a**, **b**) and the weathered soil suspension (**c**, **d**). The values represent the standard deviation of three replicates. The letters *a*, *b* and *c* indicate the statistical difference among samples

Respirometric profiles for the RCSS are presented in Fig. 4a and b. The CO_2_ production rate indicates that the maximum activity took place during the first 15 days of the culture (Fig. 4a). The CO_2_ production rate (mg C-CO_2_ kg^−1^ perlite h^−1^) presented a positive correlation between the initial load (0, 10,000 and 20,000 mg kg^−1^) of hydrocarbons and the increase in CO_2_ production rate for the two sources of inocula (Fig. 4a, c). The maximum respiration rate was recorded for the RCSS inoculum (29.16 ± 0.002 mg C-CO_2_ kg^−1^ h^−1^) on perlite with ~20,000 mg kg^−1^, while the CO_2_ production rate was twice lower (12.23 ± 1.08 mg C-CO_2_ kg^−1^ h^−1^) in the ~10,000 mg kg^−1^ experimental group (Fig. 4a). The cumulative CO_2_ production confirmed significant differences (*p* < 0.05) among the mineralisations recorded for the different hydrocarbon concentrations. A short lag stage with a low respiration rate was observed in the presence of the RCSS inoculum. Later, periods of high mineralisation were observed, indicating cyclical periods of biotransformation-oxidation. These results agree with the enzymatic profiles obtained for the same RCSS inoculum (Fig. 3), where the lipase activity recorded maximum levels during the first 10 days of culture.

Attempts to use soil respiration as a criterion to assess respiration in contaminated soils have been carried out in the past. In a study on the scale-up of chemical-biological remediation techniques applied to a highly contaminated soil, Guzmán-Osorio (2011) reported respiration levels of 0.86 to 2.6 mg C-CO_2_ kg^−1^ h^−1^ of soil after a remediation treatment. Plaza et al. (2005) obtained respiration rates of 8.6 to 38.5 mg C-CO_2_ kg^−1^ h^−1^ during the natural attenuation of hydrocarbon contaminated soils (18,900–172,700 mg kg^−1^).

Table 3 presents the residual TPH content values obtained during the biodegradation potential assay with the recently contaminated soil and the weathered soil suspension inocula on perlite as solid support. The inocula of the RCSS produced a similar hydrocarbon biodegradation (~27 %) for the two initial concentrations after 28 days of culture. Escalante-Espinosa et al. (2005) carried out a phytoremediation study with artificially contaminated perlite (5000 mg kg^−1^) and a microbial consortium as inoculum, and reported a biodegradation of 3 to 12 % for the inoculated and non-inoculated treatments (unplanted pots), respectively. In the same experiment, inoculated pots recorded a 75 % of TPH removal after 190 days of experimentation. Díaz-Ramírez et al. (2010) worked with a hydrocarbon (Olmeca crude oil) spiked clay-loam soil and a biostimulation/bioaugmentation trial (30 days of culture), and recorded biodegradation values of 21.65 and 18.09 % for inoculated and non-inoculated treatments, respectively. Zhen-Yu et al. (2012) studied contaminated clay soils (20,000 mg kg^−1^) inoculated with degrading microorganisms (1 × 10^5^ CFU kg^−1^) and reported a biodegradation level of less than 30 % after 20 days of treatment.

**Table 3:** Residual TPH content throughout the biodegradation potential assay using perlite as solid support inoculated with recently contaminated soil (RCSS) and weathered soil (WSS) suspensions

Time (days)	SSRC^a^ (mg kg^−1^)		WSS^c^ (mg kg^−1^)^b^	
0	15,000 ± 4,768	22,000 ± 1618	15,000 ± 4,769	22,000 ± 1,618
7	11,893 ± 851	19,399 ± 830	14,704 ± 805	16,283 ± 158
14	12,945 ± 2,618	16,833 ± 223	13,104 ± 592	18,834 ± 518
22	12,251 ± 649	17,134 ± 173	11,921 ± 344	15,220 ± 914
29	10,873 ± 796	16,125 ± 1595	11,563 ± 618	16,632 ± 1,570
Relative TPH loss (%)	27.51	26.70	22.91	24.39

^a^Recently contaminated soil suspension inoculum

^b^Values represent the mean of three replicates

^c^Weathered soil suspension inoculum

### Weathered Soil Inoculum

The respirometric kinetics for the WSS are presented in Fig. 4c and d. The maximum CO_2_ production rate period was registered between 7 and 12 days of culture in the presence of highly contaminated perlite. A maximum of 14.32 mg C-CO_2_ kg^−1^ h^−1^ was recorded for 10,000 g kg^−1^ of perlite, and 8.30 mg C-CO_2_ kg^−1^ h^−1^ were recorded for 20,000 g kg^−1^ of perlite (Fig. 4c). These values are twice lower than those recorded for the RCSS. Respiratory activity later decreased to almost 3.11 mg C-CO_2_ kg^−1^ h^−1^ in the last week of the culture. The cumulative CO_2_ production presented a long adaptation stage (first 7 days), after which respiration abruptly increased to around 1470 mg C-CO_2_ kg^−1^ (20,000 mg kg^−1^), 1184.4 mg C-CO_2_ kg^−1^ (10,000 mg kg^−1^) and 360 mg C-CO_2_ kg^−1^ (non-contaminated perlite) (Fig. 4d). These results are significantly lower (*p* < 0.05) than those recorded for the RCSS. Additionally, more respiration peaks were recorded for the bottles inoculated with RCSS (Fig. 4a, c). Despite these results, respiration in the WSS inoculum was greater than that previously reported for this type of tropical soil (Guzmán-Osorio 2011). These findings indicate that the RCSS has a high capacity to grow on TPH typically composed of 67 % aliphatics, 24 % aromatics and 9 % polars and asphaltenes (Díaz-Ramírez et al. 2003; Gallegos Martínez et al. 2000). In the case of the weathered soil, the lower initial response may reflect an adaptation to a more complex and recalcitrant type of carbon source (such as aged crude oil residues). Based on the relative abundance and the different colony morphology, a greater microbial diversity was observed in the samples inoculated with the RCSS, in comparison with the perlite added to the WSS where less than five predominant morphological types were detected growing on TSA.

It is generally assumed that even if CO_2_ production is not a direct measure of the volume of oil carbon biotransformation, changes recorded in the microbial activity in oil-contaminated environments reflect a microbial decomposition of the oil (Oh et al. 2002).

Bending et al. (2004) examined the interrelationships among functional biochemical and microbial soil quality indicators, and reported a CO_2_ respiration rate of 41 to 52 mg C-CO_2_ kg^−1^ h^−1^ for agricultural soils under different management programmes.

Statistical analyses (multifactorial ANOVA) found significant differences in CO_2_ production (*p* < 0.05) for the factors (a) contamination source (contamination history), (b) hydrocarbon concentration and (c) sampling time. With respect to the soil inocula, the analyses obtained significant differences and indicated that the RCSS inoculum presented the greatest cumulative CO_2_ production.

## Conclusion

The evaluation of biological indicators and of the biodegradation potential in soils with different contamination histories made it possible to determine a high background microbial activity in these highly contaminated soils. Lipase activity and respiration rates were the best biological indicators, with greater levels than those recorded in non-contaminated soils. The biodegradation efficiency assessing approach based on the estimation of biological indicators (lipase and dehydrogenase activities, and respirometric profiles), in a model system under hydrocarbon bioavailability conditions, showed that the recently contaminated soil was the most active. The profiles of lipase and dehydrogenase microbial activity indicated a highly oxidative initial stage, even duplicating the levels of produced CO_2_ with respect to the weathered soil inoculum. A positive correlation was obtained for hydrocarbon biodegradation and lipase activity in the two inocula. Samples from the recently contaminated site presented a high potential for the microbial biodegradation of the hydrocarbons spilled at the site. The protocols developed in this study may help to characterise contaminated sites, apply monitored natural attenuation programmes and select bioremediation strategies to be applied to weathered and recently contaminated tropical soils.
